# Actuarial senescence in a dimorphic bird: different rates of ageing in morphs with discrete reproductive strategies

**DOI:** 10.1098/rspb.2018.2053

**Published:** 2018-12-05

**Authors:** Melissa L. Grunst, Andrea S. Grunst, Vincent A. Formica, Marisa L. Korody, Adam M. Betuel, Margarida Barcelo-Serra, Rusty A. Gonser, Elaina M. Tuttle

**Affiliations:** 1 Department of Biology, Indiana State University, Terre Haute, IN 47809, USA; 2 Department of Biology, Behavioural Ecology and Ecophysiology Group, University of Antwerp, 2610 Wilrijk, Belgium; 3 Department of Biology, Swarthmore College, Swarthmore, PA 19081, USA; 4 San Diego Zoo Institute for Conservation Research, San Diego, CA 92101, USA; 5 Atlanta Audubon Society, Atlanta, GA 30342, USA

**Keywords:** actuarial senescence, reproductive strategies, sexual selection, white-throated sparrow

## Abstract

It is often hypothesized that intra-sexual competition accelerates actuarial senescence, or the increase in mortality rates with age. However, an alternative hypothesis is that parental investment is more important to determining senescence rates. We used a unique model system, the white-throated sparrow (*Zonotrichia albicollis*), to study variation in actuarial senescence. In this species, genetically determined morphs display discrete mating strategies and disassortative pairing, providing an excellent opportunity to test the predictions of the above hypotheses. Compared to tan-striped males, white-striped males are more polygynous and aggressive, and less parental. Tan-striped females receive less parental support, and invest more into parental care than white-striped females, which are also more aggressive. Thus, higher senescence rates in males and white-striped birds would support the intra-sexual competition hypothesis, whereas higher senescence rates in females and tan-striped birds would support the parental investment hypothesis. White-striped males showed the lowest rate of actuarial senescence. Tan-striped females had the highest senescence rate, and tan-striped males and white-striped females showed intermediate, relatively equal rates. Thus, results were inconsistent with sexual selection and competitive strategies increasing senescence rates, and instead indicate that senescence may be accelerated by female-biased parental care, and lessened by sharing of parental duties.

## 1. Introduction

Actuarial senescence occurs when mortality rate increases with age, and may reflect declines in somatic and physiological condition that make individuals more susceptible to environmental challenges [1–4]. Senescence has now been demonstrated in a wide range of natural populations [3], and evolutionary and life-history theory offers explanations for differences in ageing rates [5–7]. Yet factors underlying variation in patterns of senescence remain unclear. It is often proposed that intense sexual selection and intra-sexual competition accelerate rates of senescence, which may result in sex differences in rates of ageing [8–14]. Males usually benefit more than females by pursuing multiple mating partners, and are thus often subject to higher levels of sexual selection and intra-sexual competition [15,16]. Sexually selected traits involved in intra-sexual competition, such as large body size and elaborate ornaments, may be costly to produce and maintain, promoting the evolution of faster senescence. Thus, males are often predicted to adopt a live-fast, die-young reproductive strategy, and to age at faster rates than females [9,17]. The steroid testosterone may support high levels of intra-sexual competition, particularly in males, and deleterious effects of this hormone have been proposed to mediate higher rates of senescence in males [9]. Higher levels of intra-sexual competition are especially expected to promote the evolution of more rapid senescence when intense competition for mates enforces lower reproductive success among older individuals, which may be in suboptimal condition and unable to efficiently compete [8].

However, high levels of intra-sexual competition need not always favour a fast pace of life and accelerated actuarial senescence. Indeed, intense sexual selection on condition and performance may have pleiotropic effects that lead to increased longevity [9,18]. In addition, where reproductive success increases with age due to mate choice dynamics [19], experience or social status [20], sexual selection is not expected to greatly increase ageing rates [21]. Empirical results lend equivocal support to the hypothesis that highly competitive reproductive strategies, for instance in males relative to females, are costly and associated with higher rates of senescence [11,22–28].

As an alternative to intra-sexual competition, levels of parental investment may be more predictive of the rate at which senescence progresses. The higher rates of senescence sometimes reported for males of polygamous species, especially mammals, may reflect high levels of intra-sexual competition [8,29,30]. However, in species with more monogamous social systems sex differences in senescence are generally predicted to be smaller, and sex-specific patterns of senescence may reflect sex-biases in parental care, rather than the intensity of intra-sexual competition [22,27]. In birds, parental care and mortality rates are often female-biased [14,22]. Thus, the relative importance of intra-sexual competition versus parental investment in determining rates of senescence remains unclear.

Species with alternative morphs that display discrete reproductive strategies offer a unique opportunity to study how reproductive strategies affect patterns of senescence. In species with alternative male reproductive morphs, one morph is often more ‘female-like’, and adopts a less competitive reproductive strategy. For instance, in the ruff (*Philomachus pugnax*) males occur as one of three alternative morphs, one of which mimics females in size and plumage characteristics, and engages in sneak copulation [31,32]. If intra-sexual competition accelerates senescence, less competitive morphs are predicted to senesce at slower rates than more competitive morphs. Little empirical work has explored the possibility for morph-specific patterns of senescence despite the powerful potential of such work to test theories of ageing (but see [33]).

We used a species that displays genetically determined morphs in both sexes, the white-throated sparrow (*Zonotrichia albicollis*), to study variation in actuarial senescence. The morphs of the white-throated sparrow display discrete reproductive strategies and disassortative pairing, providing an excellent opportunity to evaluate the contribution of intra-sexual competition and parental care to determining ageing rates [34–36]. Morph is genetically determined by a large rearrangement on the second chromosome, which is thought to function as a supergene, and the morphs share the same habitat [36]. White-striped males (WMs) pursue extra-pair copulation, are more aggressive, and sing at higher rates than tan-striped males (TMs), which invest more into paternal care [37–41]. WMs also have higher levels of testosterone during the breeding season than TMs [42–45]. Similar to males, white-striped females (WFs) are more aggressive than tan-striped females (TFs), participating in territorial defence and sometimes singing. In addition, disassortative pairing by morph means that social pairs consist of either WMs and TFs (W × T pairs), or TMs and WFs (T × W pairs), almost exclusively [37,40]. Parental care is female-biased in W × T pairs, with TFs receiving relatively little paternal support. In T × W pairs, care for offspring is more cooperative and biparental [38,40].

Thus, given a strong effect of intra-sexual competition on rates of senescence, one would predict higher senescence in WMs relative to other morph–sex classes, unless male reproductive success increases with age [9]. In our population, the reproductive success of WMs shows a negative quadratic relationship with age, whereas the reproductive success of TMs linearly increases with age [46]. Thus, given a strong role for intra-sexual competition in increasing rates of ageing, we predicted higher rates of actuarial senescence and reduced longevity in WMs relative to other morph–sex classes. If competitive behaviour contributes to somatic declines and accelerated ageing, we also expected faster rates of senescence in WFs relative to TFs. On the other hand, if the demands of parental investment have a strong effect on senescence [22], we predicted higher senescence rates in tan-striped birds of both sexes, with this effect particularly pronounced in TFs, which, because of social dynamics, receive little parental support. In combination with recent work on reproductive senescence in this system [46], this study provides intriguing new insight into the processes underlying senescence in a wild vertebrate population.

## 2. Methods

### (a) Study site and field methods

We used a long-term dataset derived from a population of white-throated sparrows breeding in the vicinity of Cranberry Lake Biological Station (State University of New York, College of Environmental Science and Forestry; 44°15′ N, 74°48′ W). The dataset spans 19 years, from 1998 to 2016, and includes 158 WMs, 147 TMs, 108 WFs and 127 TFs. Birth years were known for 59 individuals (20 WMs, 16 TMs, 13 WFs and 10 TFs), and death years for 510 individuals (149 WMs, 139 TMs, 100 WFs and 122 TFs). We banded adult birds and nestlings (day 6) with Fish and Wildlife bands bearing unique identification numbers and with colour band combinations that allow identification in the field (Master Banding Permit 22296 to E.M.T.). Banding of nestlings allowed recruits to be monitored throughout their reproductive lifespan. In each year following initial banding, individuals were identified via colour bands in the field or upon recapture in mist nets. Each year we comprehensively surveyed the study site to ensure that all breeding individuals were re-sighted. We could not definitely determine the fate of juveniles in our population due to high juvenile dispersal rates. Thus, we only included individuals that survived to their first breeding season in our model. As a result, survival probability in the first time step (from age 0 to 1) is equal to 1 for our analysis.

### (b) Bayesian survival trajectory analysis

To quantify age-specific survival across the morph–sex classes, we used R package BaSTA (Bayesian Survival Trajectory Analysis) to perform capture–mark–recapture (CMR) analysis under a Bayesian framework [47]. This method copes with two common problems of field studies on wild populations: (1) unknown birth and death dates (left-truncated and right-censored data); and (2) low recapture probabilities (not an issue in our study). Survival estimates are adjusted to recapture probabilities, and missing times of birth and death are estimated from the population mean [47,48]. BaSTA attributes an age to individuals with unknown age through comparison of their survival parameters with individuals of known age. As a result, individuals of unknown age contribute relatively little to the precision of survival parameters. Thus, we acknowledge that the relatively low number of individuals with known birth years in our dataset (reflecting the challenges of this field study) presents some limitation. Nevertheless, meaningful patterns emerged despite this limitation, and there is no reason to expect that it biased our conclusions.

We applied BaSTA to optimize parametric mortality (survival) functions using Markov chain Monte Carlo (MCMC) algorithms. The mortality or hazard function describes how the risk of mortality changes with age, and is defined as 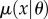, where *x* corresponds to age and 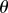 is a vector of mortality parameters to be estimated. We tested which of three commonly applied mathematical functions best-described patterns of actuarial senescence in the white-throated sparrow: (1) the Weibull function, (2) the Gompertz function and (3) the exponential function [49]. The Weibull function is a power function that assumes independence of baseline and age-related mortality, and has been commonly used to describe patterns of senescence in wild and captive bird populations. Age-related mortality in the Weibull function is determined by the equation: 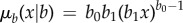, where *b*_0_, *b*_1_ > 0, b_0_ is the Weibull shape parameter and *b*_1_ is the scale parameter [47,49]. In the Weibull model, the value of *b*_0_ determines the shape of the mortality function, which can show an accelerating increase (*b*_0_ > 2), a decelerating increase (1 > *b*_0_ > 2), a decrease (0 < *b*_0_ < 1) or constant mortality (*b*_0_ = 1), whereas *b*_1_ affects the magnitude of the senescence rate. The Gompertz function is an exponential function in which age-related mortality is scaled by baseline mortality on an age-specific basis. Age-related mortality in the Gompertz function is determined by the equation: 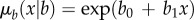, where −∞ < *b*_0_, *b*_1_ < ∞ [46]. In the Gompertz function, *b*_0_ is baseline mortality and *b*_1_ determines the pattern of age-dependent mortality. If *b*_1_ > 0 mortality increases exponentially with age, if *b*_1_ < 0 mortality decreases exponentially with age, and if *b*_1_ = 1 mortality is constant across age classes_._ In the exponential function, no age-dependent mortality occurs, and the function is defined by the formula: 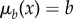, where *b* > 0. We also tested three different versions of the Weibull and Gompertz function by specifying a simple, Makeham or bathtub shape. Specifying a Makeham structure ensures modelling of finite mortality by adding a constant to the function and making the model converge to the parameter *c*, rather than 0, as age increases. The bathtub structure provides for concave mortality functions (decreasing mortality at early ages) by adding a declining Gompertz term and a constant to the basic mortality function [47]. We included morph–sex class (WM, TM, WF, TF) as a categorical covariate, modelled as a linear function of the survival parameters (using the *fused* covariate structure option).

We ran MCMC optimizations using four parallel simulations with 50 000 iterations and a 5000 burn in period (number of MCMC steps to be discarded at the beginning of the simulation), and thinning set to 100 (minimizes serial autocorrelation). We compared the fit of the seven possible mortality functions based on the lowest deviance information criterion (DIC) [47,50]. The Weibull model with a bathtub shape best fitted our data. Thus, we compared the posterior distributions of the mortality parameters included in the bathtub Weibull function using the Kullback–Leibler divergence calibration (KLDC) [51,52] included in BaSTA. KLDC reflects the probability of values deriving from one distribution coming from a second distribution. KLDC = 0.5 when distributions are identical, and KLDC = 1 when distributions do not overlap. KLDC values greater than 95% are conventionally interpreted as reflecting a significant difference.

We also generated life tables in BaSTA for each morph–sex class, and compare estimated remaining lifespan at age 2 [4].

## 3. Results

The Weibull function with a bathtub shape provided the best fit to our data (electronic supplementary material, table S1), so we report the results of simulations based on this model. The model including morph–sex type as a covariate was not as well supported (DIC: 9605) as the model including only sex as a covariate (DIC: 9569), the model including only morph as a covariate (DIC: 9576) or the baseline model without any covariate (DIC: 9578). The model including sex alone was the best-fitting model and had a lower DIC score than the baseline model (ΔDIC: 9), whereas the model including morph alone had a slightly lower DIC score than the baseline model (ΔDIC: 2). However, the DIC score of the model including morph–sex type is calculated with all of the five bathtub model parameters calculated independently, whereas only the Weibull scale parameter (*b*_1_) differed between the groups (see below). As a result, the DIC score of the morph–sex model is penalized for unnecessary parameters (this is also true for the models with sex or morph alone included as a covariate, but to a lesser extent than the model with morph–sex type). Thus, we report results of the model with sex alone, morph alone, and all four morph–sex classes included.

In all cases, model parameters converged appropriately, as assessed by the Gelman–Rubin criterion (potential scale reduction factors close to 1) [53], and displayed low serial autocorrelations (less than 0.05). Recapture probability was 94.8% (95% CI: 93.2–96.3%) for all morph–sex classes combined, and was similar across morph and sex classes. Recapture probabilities were 92.7% (CI: 89.7–95.3%), 89.0% (CI: 84.9–92.4%), 87.6% (CI: 82.5–91.9%) and 87.4% (CI: 82.3–91.7%) for WMs, TMs, WFs and TFs, respectively.

For both sexes (electronic supplementary material, table S2), both morphs (electronic supplementary material table S3) and each morph–sex class (table 1), the Weibull shape parameter were between 1 and 2 (1 < *b*_0_ < 2), indicating a decelerating increase in mortality rate with age. Thus, actuarial senescence occurred in our system, and followed a similar pattern across morph–sex classes, because *b*_0_ did not differ depending on sex or morph (electronic supplementary material, tables S2 and S3; table 1). The bathtub parameters included in the model also did not differ between morph–sex classes (table 1).

**Table 1.** Coefficient estimates, 95% credible intervals and Kullback–Leiber discrepancy calibration values (KLDC) for BaSTA model parameters for the model with all four morph–sex types. *a*_0_ and *a*_1_ = bathtub parameters, *c* = Makeham parameter, *b*_0_ = Weibull shape parameter, *b*_1_ = Weibull scale parameter. Bold KLDC values indicate significant (greater than 95%) differences in posterior distributions.

			KLDC		
	estimate	95% CI	TM	WF	WM
*a*_0_ TF	−3.633	−5.059, −2.445	0.503	0.512	0.505
*a*_0_ TM	−3.694	−5.101, −2.573		0.523	0.500
*a*_0_ WF	−3.498	−4.813, −2.430			0.529
*a*_0_ WM	−3.719	−5.068, −2.633			
*a*_1_ TF	0.909	0.693, 0.037	0.501	0.500	0.500
*a*_1_ TM	0.932	0.725, 0.031		0.500	0.504
*a*_1_ WF	0.944	0.688, 0.039			0.502
*a*_1_ WM	0.880	0.678, 0.035			
*c* TF	0.023	0.001, 0.089	0.511	0.509	0.519
*c* TM	0.021	0.001, 0.076		0.540	0.501
*c* WF	0.026	0.001, 0.095			0.554
*c* WM	0.020	0.004, 0.071			
*b*_0_ TF	1.973	1.704, 2.284	0.593	0.530	0.746
*b*_0_ TM	1.880	1.613, 2.165		0.528	0.559
*b*_0_ WF	1.921	1.614, 2.269			0.642
*b*_0_ WM	1.816	1.581, 2.086			
*b*_1_ TF	0.354	0.313, 0.396	**0.987**	0.912	**1**.**000**
*b*_1_ TM	0.299	0.262, 0.338		0.612	0.928
*b*_1_ WF	0.313	0.267, 0.358			**0**.**974**
*b*_1_ WM	0.263	0.227, 0.296			

**Figure 1.**
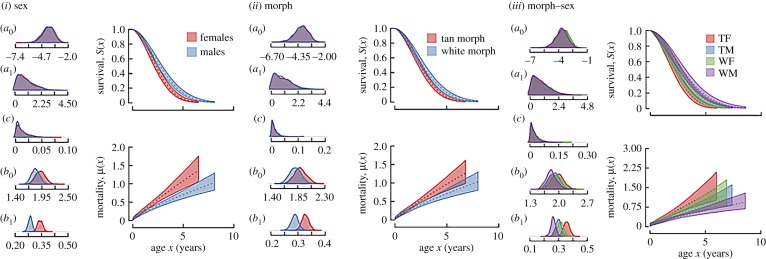
Estimated age-dependent survival probability and mortality hazards for the (*i*) sexes, (*ii*) morphs and (*iii*) four morph–sex classes from a Weibull model with a bathtub shape. Coloured regions show 95% credible intervals. Posterior distributions for the bathtub parameters (*a*_0_ and *a*_1_), Makeham parameter (*c*), Weibull shape parameter (*b*_0_), and Weibull scale parameter (*b*_1_) are shown to the left. (Online version in colour.)

However, there were significant differences in the Weibull scale parameter (*b*_1_), indicating differences in the rate of senescence between the morph–sex classes. In the best-fitting model including sex as a covariate, males and females differed in the Weibull scale parameter (*b*_1_), with males having a significantly lower Weibull scale parameter (*b*_1_) than females (electronic supplementary material, table S2; figure 1*i*). In the model including morph as a covariate, white-striped individuals had a lower Weibull scale parameter (*b*_1_) than tan-striped individuals (electronic supplementary material, table S3; figure 1*ii*). In the model including morph–sex type as a covariate, TFs displayed the highest Weibull scale parameter (*b*_1_) and WMs the lowest scale parameter. The scale parameter of TMs and WFs showed intermediate values (table 1 and figure 1*iii*). The scale parameter of TFs was higher than that of WMs and TMs, and tended to be higher than that of WFs. Compared to WMs, WFs had a higher scale parameter, and TMs tended to have a higher scale parameter (table 1 and figure 1*iii*). TMs and WFs did not differ in the scale parameter.

**Figure 2.**
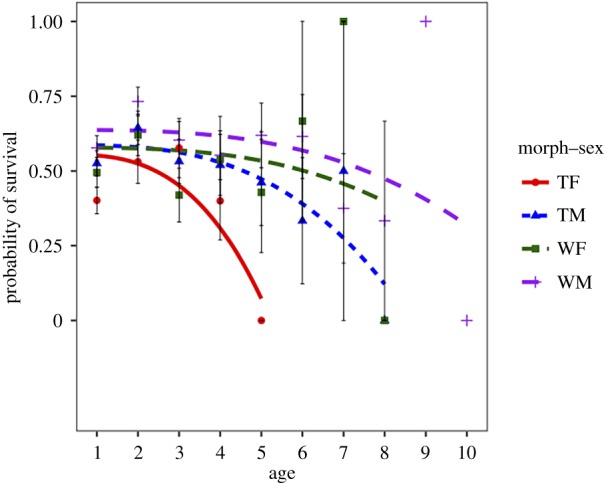
Probability of survival and associated standard error at each age for the four morph–sex classes. (Online version in colour.)

Life tables for the four morph–sex classes are presented in table 2, and the probability of death at each age is plotted in figure 2. Life expectancy at age 2 relative to WMs was 34.8% less in TFs, 21.2% less in WFs and 16.2% less in TMs.

**Table 2.** Life tables for the four morph–sex classes: tan females (TF), white females (WF), tan males (TM) and white males (WM). *n_x_* = number alive at start of interval, *l_x_* = proportion surviving at start of interval, *d_x_* = number of deaths in interval, *q_x_* = death rate in interval, *e_x_* = remaining life expectancy at end of interval.

age	*n_x_*	*l_x_*	*d_x_*	*q_x_*	*e_x_*	age	*n_x_*	*l_x_*	*d_x_*	*q_x_*	*e_x_*
**TF**						**WF**					
1–2	127	1.0	73	0.574	1.405	1–2	106	1	50	0.471	1.698
2–3	54	0.425	23	0.425	1.629	2–3	56	0.528	19	0.339	1.767
3–4	31	0.244	11	0.354	1.467	3–4	37	0.349	18	0.486	1.418
4–5	20	0.157	12	0.6	1	4–5	19	0.179	11	0.578	1.289
5–6	8	0.062	6	0.75	0.75	5–6	8	0.075	5	0.625	1.375
6–7	2	0.015	2	1	0.5	6–7	3	0.028	1	0.333	1.833
						7–8	2	0.018	0	0	1.5
						8–9	2	0.018	2	1	0.5
**TM**						**WM**					
1–2	144	1	66	0.458	1.805	1–2	154	1	63	0.409	2.155
2–3	78	0.541	26	0.333	1.910	2–3	91	0.590	23	0.252	2.302
3–4	52	0.361	21	0.403	1.615	3–4	68	0.441	22	0.323	1.911
4–5	31	0.215	16	0.516	1.370	4–5	46	0.298	23	0.5	1.586
5–6	15	0.104	7	0.466	1.3	5–6	23	0.149	9	0.391	1.673
6–7	8	0.055	5	0.625	1	6–7	14	0.090	6	0.428	1.428
7–8	3	0.020	2	0.666	0.833	7–8	8	0.051	5	0.625	1.125
8–9	1	0.006	1	1	0.5	8–9	3	0.019	2	0.666	1.166
						9–10	1	0.006	0	0	1.5

## 4. Discussion

Our results suggest that actuarial senescence occurs in the white-throated sparrow, with the Weibull model, in which mortality hazards are modelled as a power function of age, fitting the data substantially better than the exponential model, in which mortality is age-independent, or the Gompertz model, in which mortality increases as an exponential function of age. The Weibull model with a bathtub shape provided a better fit to the data than the model with a simple or Makeham shape, suggesting that early mortality patterns may be governed by a different process than senescence. For all morph–sex classes, the Weibull shape parameter was between 1 and 2, suggesting a similar pattern of senescence characterized by a decelerating increase in mortality rate with age. However, we found support for different rates of senescence between the sexes and morphs, as indicated by differences in the Weibull scale parameter. The biggest difference in senescence rate was between the sexes, rather than between the morphs, suggesting that morph differences in physiology and behaviour have less effect on the process of senescence than differences between the sexes. Nevertheless, there was some evidence for an effect of morph on the ageing process. The differences suggested between both the sexes and morphs provide insight into the relative effects of intra-sexual competition and parental care on the process of senescence.

Our results did not support the hypothesis that intense intra-sexual competition promotes investment in reproductive success at the expense of survivorship, such that male (or female) phenotypes that invest more heavily in sexual competition should generally suffer more rapid ageing [9]. Males, the more competitive sex, displayed a lower rate of senescence than females, and the more competitive and aggressive white-striped morph a lower senescence rate than the tan-striped morph. Moreover, when analysed on the level of the morph–sex class, WMs, which compete more intensely for mates than TMs (as demonstrated by high territorial aggressiveness, testosterone concentrations during breeding [43,44], singing rates and rates of extra-pair copulation [40,54]), had a lower senescence rate than both WFs and TFs, and tended to have a lower senescence rate than TMs. Thus, results on the levels of sex, morph and morph–sex class are all inconsistent with highly competitive strategies being associated with higher senescence rates.

Rather, our results are consistent with the hypothesis that higher levels of parental investment increase ageing rates [22]. Females had a higher rate of senescence than males, and as in many species of birds, female white-throated sparrows alone incubate the eggs. Incubation represents a substantial parental investment and may contribute to increasing rates of senescence in females relative to in males. Although to a lesser extent, the tan-striped morph also displayed a higher senescence rate than the white-striped morph, and tan-striped white-throated sparrows of both sexes have been observed to be more parental than white-striped counterparts [36,38–40]. On the level of morph–sex class, TFs had the fastest rate of senescence, and our past work also demonstrates fast rates of reproductive senescence in TFs [46]. TFs perform parental duties with little support from their white morph mates, and unsupported motherhood can have substantial costs [55]. Thus, given high somatic costs of parental care, the highest costs are expected in TFs. TFs may also have difficulty sustaining reproductive success as they age due to lack of parental support, dampening selection for late-life performance and favouring the evolution of faster rates of senescence [46].

Sharing parental duties may dampen the somatic costs of parental care and thereby reduce rates of actuarial senescence relative to that experienced by unsupported carers. Indeed, in cooperative breeding species, the presence of helpers at nests has been demonstrated to lower physiological costs of reproductive effort [56]. In the support of reduced costs of parental care in cooperative systems, TMs and WFs, which share parental care relatively equally, showed very similar patterns of actuarial senescence to each other, with rates intermediate between the low rate of white males and high rate of TFs.

Furthermore, although the competitive behaviours and conspicuous displays associated with sexual selection are often proposed to increase mortality risk in males [9,57], parental effort, particularly when unsupported, may also elevate mortality risk. For instance, females (or males in some species) may be prone to depredation when incubating nests, provisioning nestlings or defending offspring [58–60], especially if not warned or otherwise supported by their mates or other conspecifics. Indeed, in eiders (*Somateria mollissima*), which exhibit uniparental female care, high predation risk among care-giving females leads to facultative formation of cooperative care-giving coalitions [61]. High mortality risk in ageing and unsupported TFs providing parental care could accelerate rates of actuarial senescence.

Notably, there was a larger sex difference in age-dependent mortality rate in W × T pairs relative to T × W pairs, which is consistent with past work suggesting that sex differences in senescence are more pronounced in more polygamous breeding systems [8,29,30]. However, in white-throated sparrows the sex difference is in the opposite direction than expected given a high cost of intense male mating effort, and instead may reflect differences in parental costs. Owens & Bennett [22] suggested that sex differences in parental care, rather than the competition over mates, may drive sex-biased mortality patterns in birds. Our findings suggest that this might also be true for patterns of actuarial senescence in bird species that exhibit extensive parental care.

We recently demonstrated differences in reproductive senescence between the morph–sex classes of the white-throated sparrow that contrast in some respects to our findings for actuarial senescence. When combined with the current results, our past findings regarding reproductive senescence further inform our understanding of mechanisms of senescence in this species. For females, reproductive senescence differed between the morphs with the reproductive success of TFs linearly declining with age, and that of WFs showing a negative quadratic relationship [46]. Thus, as for patterns of actuarial senescence, patterns of reproductive senescence in females are consistent with the hypothesis that higher parental demands in TFs may accelerate rates of ageing. However, in males we found faster rates of reproductive senescence in WMs than in TMs. The reproductive success of WMs showed an eventual senescent decline with age (negative quadratic relationship), whereas that of TMs steadily increased [46]. Thus, this pattern is not consistent with the pattern of actuarial senescence. Consequently, high rates of reproductive success among old WMs cannot explain the trend towards lower rates of actuarial senescence in WMs relative to TMs, and are unlikely to explain the difference between males and females.

A possible explanation for the disconnect between patterns of actuarial and reproductive senescence in male is that these two types of ageing are determined by different processes. Specifically, patterns of reproductive senescence may be more strongly affected by intra-sexual competition than are patterns of actuarial senescence, because especially in males, reproductive success often entails direct competition over mating opportunities. Thus, the high rate of reproductive senescence in WMs might reflect the difficulty of sustaining a highly competitive reproductive strategy as ageing progresses, rather than a high rate of somatic senescence [46]. By contrast, the low rate of actuarial senescence in WMs could reflect a low rate of somatic senescence, which could result from lower parental demands. Also, sexual selection on WMs could also lead to positive pleiotropic effects on survivorship and somatic maintenance [9], lowering rates of actuarial senescence without necessarily allowing WMs to maintain high rates of reproductive success at old ages.

As a caveat, differences in actuarial senescence between the morph–sex classes could arise from any number of morph or sex-linked traits, especially given a large number of genes implicated in the rearrangement on chromosomes 2. Conclusively demonstrating a causative effect of any one variable, such as mating or parental effort, would require experimental manipulation, or a meta-analysis approach. Nevertheless, although the exact source of causation awaits further study, we can eliminate certain contingencies, such as the proposition that sexual selection induces faster rates of actuarial senescence in white males. Moreover, we can assess whether our data is consistent or inconsistent with previously proposed theory, as done above.

In conclusion, our data suggest differences in the rate of actuarial senescence between white-throated sparrow morph–sex classes with discrete reproductive strategies. Specifically, TFs, which exhibit high levels of relatively unassisted parental care, displayed the fastest rate of actuarial senescence, whereas highly competitive WMs displayed the lowest rate. These results suggest that parental care may entail higher somatic costs than sexual selection and intense competition, and may thus drive patterns of actuarial senescence in this and similar species.

## Supplementary Material

Actuarial senescence in a dimorphic bird: different rates of aging in morphs with discrete reproductive strategies

## Data accessibility

Data available from the Dryad Digital Repository: http://dx.doi.org/10.5061/dryad.bq8n45v [62].
