# Supplementary material for: Actuarial senescence in a dimorphic bird: different rates of ageing in morphs with discrete reproductive strategies
Source: Proc Biol Sci. 2018 Dec 5;285(1892):20182053. doi: 10.1098/rspb.2018.2053 (PMC6283936; doi:10.1098/rspb.2018.2053)
Supplement: Actuarial senescence in a dimorphic bird: different rates of aging in morphs with discrete reproductive strategies [file rspb20182053supp1.docx]

**Online Supplementary Material**

**Article: Actuarial senescence in a dimorphic bird: different rates of aging in morphs with discrete reproductive strategies**

Grunst, Melissa L.^1,2^, Grunst, Andrea S. ^1,2^, Formica, Vincent A.^1,3^, Korody, Marisa L.^1,4^, Betuel, Adam M.^1,5^, Barcelo-Serra, Margarida^1^, Gonser, Rusty A.^1**^, Tuttle, Elaina M.^1^

^1^Department of Biology, Indiana State University, Terre Haute, Indiana, 47809

^2^Department of Biology, Behavioural Ecology and Ecophysiology Group, University of Antwerp, 2610 Wilrijk, Belgium

^3^Department of Biology, Swarthmore College, Swarthmore, Pennsylvania, 19081

^4^San Diego Zoo Institute for Conservation Research, San Diego, California, 92101

^5^Atlanta Audubon Society, Atlanta, Georgia, 30342

**Caption:** This online supplement contains tables in support of the analysis reported in the paper, “Actuarial senescence in a dimorphic bird: different rates of aging in morphs with discrete reproductive strategies”. In this manuscript, we used Bayesian survival trajectory analysis (R package BaSTA) to compare rates of actuarial senescence (increases in the mortality rate with age) between the four morph-sex classes of the dimorphic white-throated sparrow (*Zonotrichia albicollis*). The morphs of *Z. albicollis* displays discrete mating strategies and disassortative pairing. Birds of the white-striped morph, particularly males, are more aggressive and seek multiple matings, whereas tan-striped birds are more parental. Thus, this system provides an excellent opportunity to test how intra-sexual competition versus parental care contribute to aging rates. Aging rates are predicted to be higher in males and white-striped birds if intra-sexual competition exerts a stronger effect on senescence, but are predicted to be higher in females and tan-striped birds if parental care has relatively more effect.

In this paper, we tested which of three commonly applied mathematical functions best described patterns of actuarial senescence in the white-throated sparrow: (1) the Weibull function, (2) the Gompertz function, and (3) the exponential function (see paper for details on differences between models). We also tested three different versions of the Weibull and Gompertz function by specifying either a simple, Makeham, or bathtub shape. Table S1 shows the results of a model selection procedure, which was conducted to determine which type of model to use in the primary survival analysis. The Weibull model with a bathtub shape was selected (lowest DIC score).

Table S2 and Table S3 show the results of the survival analysis using sex as a covariate (Table S2) and morph as a covariate (Table S3). These analyses were run in addition to using morph-sex type as a covariation (Table 1 in the paper), and support the same conclusions.

**Table S1.** Model selection for the 7 models run in BaSTA based on deviance information criterion (DIC).

| **Model** | **Shape** | **D_ave_** | **D_mode_** | **pD** | **K** | **DIC** | **ΔDIC** |
| --- | --- | --- | --- | --- | --- | --- | --- |
| Weibull | bathtub | 9541 | 9478 | 63.8 | 21 | 9605 | 0.00 |
| Weibull | simple | 9547 | 9485 | 62.3 | 9 | 9610 | 4.65 |
| Weibull | Makeham | 9569 | 9505 | 63.9 | 13 | 9633 | 28.21 |
| Gompertz | simple | 9760 | 9704 | 56.9 | 9 | 9817 | 212.14 |
| Gompertz | bathtub | 9774 | 9714 | 60.7 | 21 | 9835 | 229.99 |
| Gompertz | Makeham | 9794 | 9739 | 54.8 | 13 | 9849 | 243.95 |
| Exponential | simple | 10173 | 10095 | 77.0 | 5 | 10250 | 644.46 |

**Table S2**. Coefficient estimates, 95% credible intervals, and Kullback-Leiber discrepancy calibration values (KLDC) for BaSTA model parameters for the model with sex as a covariate. a_0_ and a_1_ = bathtub parameters, c = Makeham parameter, b_0_ = Weibull shape parameter, b_1_ = Weibull scale parameter. Bold KLDC values indicate significant (>95%) differences in posterior distributions.

|  | Estimate | 95% CI | KLDC |
| --- | --- | --- | --- |
| a_0_ Females | -3.999 | -5.299, -2.899 | 0.512 |
| a_0_ Males | -4.128 | -5.331, -3.088 |  |
| a_1_ Females | 0.913 | 0.039, 2.568 | .500 |
| a_1_ Males | 0.929 | 0.033, 2.563 |  |
| c Females | 0.013 | 0.004, 0.045 | 0.532 |
| c Males | 0.010 | 0.003, 0.038 |  |
| b_0_ Females | 1.917 | 1.722, 2.123 | 0.723 |
| b_0_ Males | 1.815 | 1.645, 1.995 |  |
| b_1_ Females | 0.341 | 0.313, 0.369 | **0.999** |
| b_1_ Males | 0.286 | 0.263, 0.308 |  |

**Table S3**. Coefficient estimates, 95% credible intervals, and Kullback-Leiber discrepancy calibration values (KLDC) for BaSTA model parameters for the model with morph as a covariate. a_0_ and a_1_ = bathtub parameters, b_0_ = Weibull shape parameter, b_1_ = Weibull scale parameter. Bold KLDC values indicate significant (>95%) differences in posterior distributions.

|  | Estimate | 95% CI | KLDC |
| --- | --- | --- | --- |
| a_0_ Tan | -4.061 | -5.393, -3.007 | 0.500 |
| a_0_ White | -4.069 | -5.292, -3.036 |  |
| a_1_ Tan | 0.940 | 0.032, 2.634 | 0.502 |
| a_1_ White | 0.907 | 0.028, 2.526 |  |
| c Tan | 0.011 | 0.0003, 0.039 | 0.507 |
| c White | 0.013 | 0.0003, 0.042 |  |
| b_0_ Tan | 1.892 | 1.699, 2.091 | 0.658 |
| b_0_ White | 1.807 | 1.627, 1.997 |  |
| b_1_ Tan | 0.327 | 0.301, 0.353 | **0.993** |
| b_1_ White | 0.288 | 0.261, 0.315 |  |
